# Systematic Identification, Characterization, and Conservation of Adjacent-Gene Coregulation in the Budding Yeast Saccharomyces cerevisiae

**DOI:** 10.1128/mSphere.00220-18

**Published:** 2018-06-13

**Authors:** Reem S. Eldabagh, Nelson G. Mejia, Rachel L. Barrett, Christopher R. Monzo, Matthew K. So, Jonathan J. Foley, James T. Arnone

**Affiliations:** a Department of Biology, William Paterson University, Wayne, New Jersey, USA; b Department of Chemistry, William Paterson University, Wayne, New Jersey, USA; Carnegie Mellon University

**Keywords:** adjacent-gene coregulation, gene expression, Saccharomyces cerevisiae, spatial positioning

## Abstract

It is essential that cells orchestrate gene expression for the specific niche that they occupy, and this often requires coordination of the expression of large sets of genes. There are multiple regulatory systems that exist for modulation of gene expression, including the adjacent-gene coregulation of the rRNA and ribosome biogenesis and ribosomal protein families. Both gene families exhibit a nonrandom genomic distribution, often clustered directly adjacent to another member of the same family, which results in a tighter transcriptional coordination among adjacent paired genes than that of the unpaired genes within each regulon and can result in a shared promoter that coordinates expression of the pairs. This nonrandom genomic distribution has been seen in a few functionally related gene families, and many of these functional pairings are conserved across divergent fungal lineages. To date, the significance of these observations has not been extended in a systematic way to characterize how prevalent the role of adjacent-gene coregulation is in transcriptional regulation. In the present study, we systematically analyzed the transcriptional coherence of the functional pairs compared to the singletons within all gene families defined by the Gene Ontology Slim designation, using Saccharomyces cerevisiae as a model system, finding that clusters exhibit a tighter transcriptional correlation under specific contexts. We found that the longer a functional pairing is conserved the tighter its response to broad stress and nutritional responses, that roughly 25% of gene families exhibit a nonrandom genomic distribution, and that many of these clusters are conserved. This suggests that adjacent-gene coregulation is a widespread, yet underappreciated, transcriptional mechanism.

**IMPORTANCE** The spatial positioning of genes throughout the genome arrangement can alter their expression in many eukaryotic organisms. Often this results in a genomic context-specific effect on transcription. One example of this is through the clustering of functionally related genes, which results in adjacent-gene coregulation in the budding yeast Saccharomyces cerevisiae. In the present study, we set out to systematically characterize the prevalence of this phenomenon, finding the genomic organization of functionally related genes into clusters is a characteristic of myriad gene families. These arrangements are found in many evolutionarily divergent fungi and thus represent a widespread, yet underappreciated, layer of transcriptional regulation.

## Abstract (precis)

The spatial positioning of genes throughout the genome arrangement can alter their expression in many eukaryotic organisms. Often this results in a genomic context-specific effect on transcription. One example of this is through the clustering of functionally related genes, which results in adjacent-gene coregulation in the budding yeast Saccharomyces cerevisiae. In the present study, we set out to systematically characterize the prevalence of this phenomenon, finding the genomic organization of functionally related genes into clusters is a characteristic of myriad gene families. These arrangements are found in many evolutionarily divergent fungi and thus represent a widespread, yet underappreciated, layer of transcriptional regulation.

## INTRODUCTION

Cell survival depends on the proper coordination of gene expression via transcriptional regulation, integrating cues that are received to maintain homeostasis and allow proper adaptation to the environment and during development (1, 2). Gene expression is achieved through multiple means and mechanisms, which include the binding of transcription factors, the activating and repressive *trans* factors and their corresponding complexes to a gene’s promoter, and the corresponding *cis* regulatory DNA sequences (3). Often there are additional mechanisms layered on top of that, which include nucleotide modifications, alterations to chromatin—such as histone modifications and positions, as well as more complex three-dimensional subnuclear arrangements of the chromosomes within the nucleus. These changes can result in both long-term and short-term transcriptional alterations within the cell and can alter the expression of thousands of genes simultaneously (4–7).

In single-cell organisms, it is essential to balance the allocation of cellular resources and energy stores, regulating their expenditure between cell proliferation and the maintenance of homeostasis. One particularly drastic example of that is the environmental stress response (ESR) in the budding yeast Saccharomyces cerevisiae. Often this can require rapid changes to global expression levels in response to environmental perturbations during the ESR, where energy-consuming processes such as ribosome production are rapidly downregulated to allow the cell to adapt to a changing environment (2, 8, 9).

Ribosome production represents an interesting gene regulatory problem, as it involves the regulation of hundreds of genes whose protein products all must function in concert. In addition to the transcription of the ribosomal DNA (rDNA) repeats, faithful ribosome production requires transcription of both the ribosomal protein (RP) and the rRNA and ribosome biogenesis (RRB) regulons. The cell needs roughly stoichiometric levels of each of these families of genes, although the absolute levels between the RP and RRB families differ. Every ribosome needs the four rRNAs and one of each of the 79 ribosomal proteins, as well as the approximately 200 RRB gene products, which are involved in the modification and processing of the rRNA as well as the assembly of the ribosome. The RRB proteins do not remain with the ribosome after it has matured, while the RP proteins continue to remain associated with the ribosome (10). As a result, each family has evolved distinct *cis* regulatory promoter elements and *trans*-acting transcription factors. The RP family of genes contain binding sites for *Fhl1*, *Ifh1*, and *Abl1*, while the RRB family of genes contains the PAC and RRPE binding sites for *Stb3*, *Tod6*, and *Dot6* (11–15). Interestingly, it has been observed that both the RP and the RRB regulons exhibit a nonrandom genomic distribution. In both families, there is a statistically significant fraction of genes that exist as functional clusters—approximately 25% of each family. The functional clusters observed in both the RRB and the RP regulons were found primarily as gene pairs (16, 17).

Functional dissection of one of the RRB gene pairs, *MPP10-MRX12*, revealed an incidence of long-distance promoter sharing that was termed “adjacent-gene coregulation” (17). The transcription of both members of this gene pair depended on promoter elements upstream of *MPP10*, and there was a requirement for *MRX12* to be directly adjacent to *MPP10*. Physical separation by transgene insertion was sufficient to uncouple the transcriptional coregulation of *MRX12*. This transcriptional coregulation appears to be regulated in part via recruitment of histone modifications as well as the SAGA complex (17, 18). There have been documented incidences of shared promoters; however, the *MPP10-MRX12* locus was distinct for the genomic orientation and genomic distance. Most shared promoters are found between divergent genes (← →), as seen in the histone protein genes, while the *MPP10-MRX12* gene pair was found in a convergent orientation (→ ←). The distance between the transcription start sites for the genes is approximately 4 kb (17, 19).

The functional clustering of both the RRB and RP regulons resulted in a tighter transcriptional coregulation for the clustered genes compared to the singletons during induction of the ESR. In addition to these two gene families, it was observed that a number of other functionally related gene families exhibit the same, nonrandom distribution across the genome. A statistically significant fraction of the genes involved with nitrogen metabolism (NM), carbohydrate metabolism (CM), DNA damage response (DDR), heat shock response (via heat shock protein [HSP]), and toxin response (TR) can be found clustered as adjacent-gene pairs (using *P* < 0.05 as a cutoff for observing the genomic distribution). It should be noted such an arrangement was not seen in the 19 other gene families that were characterized. These functionally related clusters are conserved in widely divergent fungal lineages (20). While it has previously been reported that there are domains of correlated expression seen in S. cerevisiae, there are several outstanding questions that remain, including the role clustering plays in transcriptional coregulation outside the RRB and RP regulons and how widespread adjacent-gene coregulation is within both Saccharomyces cerevisiae and related fungi (21).

In the present study, we set out to systematically identify the effects the clustering of functionally related genes have in coordinating transcription in order to more fully characterize the importance of adjacent-gene coregulation. We report the clustering of functionally related genes in S. cerevisiae results in tighter transcriptional correlation under specific stressors, compared to the unpaired members within the same family. There is an overall tighter transcriptional response across stressors for the paired versus the unpaired members, which is conserved across divergent fungi. Furthermore, we found the greater the evolutionary distance that a gene pair is conserved correlates strongly with an increased transcriptional coherence of the pairing. Finally, we systematically characterized the genomic distribution of the 140 Gene Ontology (GO) Slim functional classifications, and we report there is a nonrandom genomic distribution seen in about 25% of gene families. This arrangement results in coordinating transcription throughout the cell cycle and is conserved throughout divergent fungi.

## RESULTS

### Functionally related gene clusters exhibit tighter transcriptional coregulation than unpaired members of the same functional set under specific transcriptional perturbations.

It has previously been observed that the rRNA and ribosome biosynthesis (RRB) and ribosomal protein (RP) regulons exhibit a nonrandom genomic distribution—approximately 25% of each regulon can be found as functional gene clusters (within each family found primarily as groups of two). This arrangement results in tighter transcription for the pairs than for the unpaired members during both the environmental stress response (ESR) and throughout the cell cycle. Aside from the RRB and the RP genes, there were a number of additional gene families that exhibited the same, nonrandom distribution across the genome but have yet to be characterized transcriptionally—including the 86 genes involved in nitrogen metabolism (NM), the 91 genes involved in carbohydrate metabolism (CM), the 175 genes involved in the DNA-damage response (DDR), the 18 heat-shock protein (HSP) genes, and the 27 toxin response (TR) genes (17, 20). To test the effect of genomic arrangement on coordinating the transcription of each family of genes, microarray gene expression profiles were extracted for each gene family, as well as the RP family (as a control), across five ESR conditions: a heat shock response, the response to methyl methanesulfonate (MMS), H_2_O_2_ stress, the transition from glucose to glycerol as a carbon source, and during nitrogen depletion. (For complete details on the data sets analyzed, refer to Materials and Methods.)

The expression profiles were plotted, and the Pearson’s correlation coefficient (PCC) was calculated for the singletons and the functionally clustered members of each gene family under every condition (Table 1; see Fig. S1 in the supplemental material). Consistent with previous analyses, we observed the functionally paired genes in the RP gene family had a higher PCC (change in PCC of >0.05) during the heat shock response (similarity score [*S*] = 0.72 versus *P* = 0.81), although during the MMS response the numbers were comparable (*S* = 0.89 versus *P* = 0.92), compared to the singletons. This most likely represents experimental differences between the studies analyzed. Likewise when we extended the RP analysis to additional environmental perturbations, we found a similar trend: there was a higher transcriptional correlation for the functionally paired genes during the transcriptional response to H_2_O_2_ stress (*S* = 0.72 versus *P* = 0.82) and the transition from glucose to glycerol as a carbon source (S = 0.51 versus *P* = 0.79). Additionally, there was a comparable level of expression seen in the RP genes during the response to nitrogen depletion (*S* = 0.82 versus *P* = 0.85) (Table 1).

**FIG 1:**
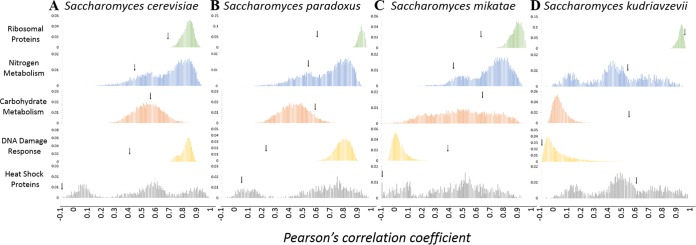
Tighter transcriptional correlation of functionally clustered genes increases with evolutionary conservation. Shown is the transcriptional correlation for every possible functional clustering arrangement that could have arisen through the use of bootstrapping with replacement. The PCC was calculated for 10,000 iterations that represent every possible combination of clustering that could have evolved (of comparable size to the actual cluster for each set), and the frequency histograms are presented for the ribosomal protein, nitrogen metabolism, carbohydrate metabolism, DNA damage response, and heat shock protein gene families (from top to bottom) in Saccharomyces cerevisiae (A), Saccharomyces paradoxus (B), Saccharomyces mikatae (C), and Saccharomyces kudriavzevii (D). The PCC for the actual clustered set of genes for each family is indicated with the arrows. For anticorrelations that have a PCC of less than −0.1, all of the values are binned at −0.1.

10.1128/mSphere.00220-18.1 FIG S1  Functionally related adjacent-gene clusters exhibit tighter transcriptional correlation during specific environmental and nutritional perturbations. Microarray transcription profiles were extracted for each gene family following perturbation and normalized to the unperturbed state (*t* = 0), and the log_2_ expression levels of every family member were plotted as a function of time. The expression was followed for a total time of either 90 min or 24 h, as indicated (with the color scheme for each family matched to the families in Fig. 1). Download FIG S1, TIF file, 1.2 MB. Copyright © 2018 Eldabagh et al. 2018 Eldabagh et al. This content is distributed under the terms of the Creative Commons Attribution 4.0 International license.

**TABLE 1:** The Pearson’s correlation coefficient of functionally related adjacent-gene clusters during environmental and nutritional perturbations

Gene family	PCC for:									
	Heat shock		MMS		H_2_O_2_		Glu→Gly		Nitrogen depletion	
	Singletons	Clusters	Singletons	Clusters	Singletons	Clusters	Singletons	Clusters	Singletons	Clusters
Ribosomal protein	0.72	0.81	0.89	0.92	0.72	0.82	0.51	0.79	0.82	0.85
Nitrogen metabolism	0.76	0.76	0.78	0.77	0.47	0.61	0.48	0.31	0.43	0.81
Carbohydrate metabolism	0.47	0.79	0.56	0.75	0.40	0.70	0.02	0.48	0.51	0.63
DNA damage response	0.59	0.71	0.00	−0.02	0.00	−0.02	0.00	0.83	0.01	0.41
Heat shock	0.61	0.31	0.48	0.75	0.49	0.33	0.11	−0.26	0.58	−0.03
Toxin response	0.48	0.66	0.89	0.92	0.29	−0.11	0.02	−0.03	0.42	0.73

We subsequently extended our analysis to each of the uncharacterized, coregulated gene families which exhibited a nonrandom genomic distribution that was previously identified (20). Each of the five families exhibited a positive PCC upon the induction of between two and five transcriptional responses. Consistent with the results seen in the RP family, we observed two families that exhibit a higher PCC for the pairs under specific conditions, such as the NM family during H_2_O_2_ stress (*S* = 0.47 versus *P* = 0.61) as well as during the response to nitrogen depletion (*S* = 0.43 versus *P* = 0.81), and the DDR family during heat shock (*S* = 0.59 versus *P* = 0.71), the transition from glucose to glycerol (*S* = 0.00 versus *P* = 0.83), and the response to nitrogen depletion (*S* = 0.01 versus *P* = 0.41). One family, the CM genes, had a higher PCC for the pairs under all five of the expression profiles analyzed. Two families, the HSP and the TR genes, had conditions under which the pairs had a higher PCC under certain conditions and a lower PCC under other conditions—and even were weakly anticorrelated in certain contexts—such as the HSP genes during the glucose-to-glycerol transition (*S* = 0.11 versus *P* = −0.26) and the toxin response genes during an H_2_O_2_ stress (*S* = 0.29 versus *P* = −0.11).

In order to determine whether there was an overall transcriptional advantage for the functionally clustered adjacent genes compared to their singleton counterparts, the composite PCC was calculated across all five of the stressors simultaneously. This analysis reveals a more complete picture of the transcriptional differences that can be seen between the functionally clustered adjacent genes compared to the singletons. Overall, each of the six functional classes of genes exhibited a positive composite PCC value across the five stress responses. For five of the six functional categories studied, there was a higher PCC value for the functionally clustered, adjacent members than for the rest of the set, with the lone outlier being the heat shock genes—which resulted in a PCC value for the adjacent members that was weakly anticorrelated compared to the rest of the set (Table 2).

**TABLE 2:** The composite Pearson’s correlation coefficient of S. cerevisiae functional clusters conserved across divergent fungal lineages

Gene family	PCC for:							
	S. cerevisiae		S. paradoxus		S. mikatae		S. kudriavzevii	
	Singletons	Clusters	Singletons	Clusters	Singletons	Clusters	Singletons	Clusters
Ribosomal protein	0.382	0.692	0.522	0.608	0.483	0.606	0.818	0.882
Nitrogen metabolism	0.314	0.466	0.332	0.552	0.267	0.442	0.252	0.539
Carbohydrate metabolism	0.199	0.579	0.244	0.611	0.228	0.657	0.14	0.525
DNA damage response	0.18	0.414	0.195	0.241	0.033	0.407	0.025	−0.625
Heat shock	0.176	−0.077	0.159	0.008	0.196	−0.306	0.251	0.602
Toxin response	0.176	0.346	NA^a^	NA	NA	NA	NA	NA

a NA, not applicable as there are no conserved functional pairings from S. cerevisiae.

### Functionally clustered pairings have a tighter transcriptional correlation across divergent fungal lineages.

The genomic positioning and spatial relationships have been conserved for many regulons across diverse fungal lineages. To characterize this effect on transcription in species other than S. cerevisiae, we expanded our analysis to look at the effect of genomic arrangement on transcriptional coregulation in the yeasts Saccharomyces paradoxus, Saccharomyces mikatae, and Saccharomyces kudriavzevii (Table 2). The composite PCC was calculated for the RP, NM, CM, DDR, and HSP genes. The TR genes were not analyzed as there were no conserved functional groupings in any of these species.

In all three yeast species, each of the gene families demonstrated an overall positive transcriptional correlation, as measured by the PCC, consistent with their behavior in S. cerevisiae. Despite the fact various degrees of conservation exist in each of the regulons, there is a stronger transcriptional correlation seen for adjacent pairings compared to the singletons seen for the RP, NM, CM, and DDR genes seen across the three species. The lone outliers are the HSP genes, where the pairings have a lower correlation in S. paradoxus and S. mikatae, comparable to the trend seen in S. cerevisiae (although they have a higher PCC value seen in S. kudriavzevii) and the DNA damage response genes, which exhibited an anticorrelation in S. kudriavzevii (*S* = 0.025 versus *P* = −0.625).

### The overall transcriptional coherence of the functionally clustered genes is positively correlated with conservation of genomic positioning.

It had been previously found that the functional groupings for the RP genes and the RRB regulons exhibited a tighter transcriptional correlation than the majority of groupings that could have evolved during the ESR (e.g., the heat shock response, hyperosmotic shock, and the response to menadione) and throughout the cell cycle (20). To extend and verify these findings, we set about to determine the significance of the composite PCC for the pairings that we observed in S. cerevisiae (Fig. 1A). A bootstrapping with replacement approach was utilized to computationally determine the PCC for all the possible functional clusters. The composite PCC across the five stress responses and nutritional perturbations analyzed above was calculated, and the frequency histograms are representative of the composite PCC for every possible pairing combination that could have occurred within each gene family. The actual PCC for each gene family is denoted by an arrow. Surprisingly, the actual pairs do not exhibit the tightest correlation compared to many of the potential groupings that could have evolved—in fact, in four gene families (the RP, NM, DDR, and the HSP genes) the actual clustered set was among the lowest calculated PCC that could have evolved. In the last two gene families, the CM genes and the TR genes (see Fig. S2 in the supplemental material), the actual clustered pairs fall roughly in the middle of the potential groupings that could have evolved.

10.1128/mSphere.00220-18.2 FIG S2  Toxin response gene family histogram. Shown is the transcriptional correlation for every possible functional clustering arrangement that could have arisen through the use of bootstrapping with replacement. The PCC was calculated for 10,000 iterations, and the frequency histograms are presented for the toxin response gene family. Download FIG S2, TIF file, 0.5 MB. Copyright © 2018 Eldabagh et al. 2018 Eldabagh et al. This content is distributed under the terms of the Creative Commons Attribution 4.0 International license.

This analysis was extended to S. paradoxus (Fig. 1B), S. mikatae (Fig. 1C), and S. kudriavzevii (Fig. 1D), which allowed for a comparison of the transcriptional effect compared to the length of conserved genomic arrangement (based on the clusters observed in S. cerevisiae). The greater the evolutionary distance where a cluster was conserved correlated with a tighter transcriptional coherence relative to every pairing that could have evolved. Indeed, in the case of the RP genes, the nitrogen metabolism genes, the carbohydrate metabolism genes, and the heat shock families, as we extended our analysis from the closely related S. paradoxus to the more distantly related S. kudriavzevii, the conserved clusters demonstrated a positive correlation with increased transcriptional coherence (that is, the greater the distance that the conservation was maintained, the greater the composite PCC compared to every possible combinatorial possibility). This result was partially true for the DNA damage response genes, which followed this trend in both S. paradoxus and S. mikatae, but not in S. kudriavzevii.

### Functional clustering is a characteristic of many gene families in Saccharomyces cerevisiae and results in tighter transcriptional correlation throughout the cell cycle.

To better gauge the extent that functionally related gene families exhibit this nonrandom genomic distribution, every functional classification designated by the Gene Ontology (GO) consortium as a GO Slim descriptor was accessed and the spatial distribution of every member was characterized (22, 23). The GO Slim designation is a standardized nomenclature (with 140 populated categories in budding yeast at the time of accession) of the most frequent categories for the classification of a gene’s function, allowing for easy classification of a gene, and it is standardized across organisms. The sizes of the categories ranged immensely, from the incredibly large nucleus (2,032 genes), cytoplasm (3,990 genes), and membrane (1,669 genes) categories to the significantly smaller translation factor activity, RNA binding (1 gene), and oligosaccharide metabolic process (2 genes) categories. The significance of the genomic distribution for each gene family was determined, and the corresponding *P* value was calculated (null hypothesis—the likelihood of the exact arrangement occurring by chance). This analysis resulted in the identification of 38 gene families that exhibited a significant *P* value of <0.05, corresponding to roughly a quarter (26%) of the families characterized (Table 3). The other 102 GO Slim categories did not cross the threshold for significance in this study (see Table S1 in the supplemental material). In order to determine the effect of this nonrandom genomic distribution on the transcription of the gene families, the PCC was calculated for the 38 gene families that exhibited the most significant, nonrandom genomic distribution (*P* < 0.05) following microarray analysis of their expression throughout the cell cycle (24). The choice of cell cycle expression, as opposed to expression throughout a stress response, was due to the wide range of molecular functions that were observed among these gene families—many of which would not respond to the ESR. Of these 38 gene families, 22 families exhibited a markedly higher PCC (change in PCC value of >0.1) for the clustered members than for the singletons (roughly 58%) (Table 3).

10.1128/mSphere.00220-18.5 TABLE S1  Functionally related gene families that exhibit a random genomic distribution in S. cerevisiae (*P* > 0.05). Download TABLE S1, DOCX file, 0.1 MB. Copyright © 2018 Eldabagh et al. 2018 Eldabagh et al. This content is distributed under the terms of the Creative Commons Attribution 4.0 International license.

**TABLE 3:** Transcriptional analysis of functionally related gene families that exhibit a nonrandom genomic distribution in S. cerevisiae

Description	GO no.	Gene set size	No. clustered	*P* value	PCC for^a^:	
					Singletons	Clusters
Vitamin metabolic process	GO:0006766	43	11	9.06E−13	0.1005	0.507
Cell wall organization or biogenesis	GO:0071554	198	38	8.35E−10	0.044	0.155
Sporulation	GO:0043934	133	20	5.09E−07	0.043	0.4626
Meiotic cell cycle	GO:0051321	282	49	8.53E−06	0.0354	0.2281
Phosphatase activity	GO:0016791	95	12	1.10E−05	0.0406	0.5193
Ribosome	GO:0005840	343	62	7.90E−05	0.1153	0.264
Structural constituent of ribosome	GO:0003735	236	35	1.01E−04	0.1667	0.0907
Monocarboxylic acid metabolic process	GO:0032787	141	17	1.29E−04	0.0523	0.5586
Amino acid transport	GO:0006865	46	4	7.34E−04	0.1148	0.2534
Lipid transport	GO:0006869	68	6	9.55E−04	0.0429	−0.1973
DNA replication	GO:0006260	139	14	2.21E−03	0.0991	0.2963
Chromatin organization	GO:0006325	318	49	2.61E−03	0.0718	0.1108
Translational initiation	GO:0006413	54	4	3.00E−03	0.1375	0.0586
Peptidase activity	GO:0008233	94	8	3.01E−03	0.1	0.3957
Pseudohyphal growth	GO:0007124	56	4	4.16E−03	0.0126	0.6038
Extracellular region	GO:0005576	31	2	4.17E−03	0.0413	−0.5761
Telomere organization	GO:0032200	78	6	4.51E−03	0.0565	0.7413
Methyltransferase activity	GO:0008168	90	7	6.10E−03	0.119	0.6294
tRNA aminoacylation for protein translation	GO:0006418	36	2	9.57E−03	0.0966	0.7101
Protein glycosylation	GO:0006486	62	4	9.69E−03	0.1917	0.6667
rRNA processing	GO:0006364	236	28	1.12E−02	0.3511	0.338
Helicase activity	GO:0004386	86	6	1.26E−02	0.1096	0.6232
Regulation of cell cycle	GO:0051726	232	27	1.28E−02	0.0411	0.047
Cytoskeleton organization	GO:0007010	238	28	1.39E−02	0.0662	0.0753
Regulation of DNA metabolic process	GO:0051052	107	8	1.54E−02	0.0584	0.4021
Nucleus organization	GO:0006997	66	4	1.60E−02	0.0906	0.1009
Cytoskeletal protein binding	GO:0008092	67	4	1.79E−02	0.0539	−0.2497
Lyase activity	GO:0016829	90	6	1.98E−02	0.0545	0.5955
Nuclease activity	GO:0004518	92	6	2.44E−02	0.0788	0.0358
DNA-templated transcription, initiation	GO:0006352	73	4	3.43E−02	0.106	0.3507
Hydrolase activity, acting on glycosyl bonds	GO:0016798	47	2	3.88E−02	0.0515	−0.0869
Cofactor metabolic process	GO:0051186	179	16	4.09E−02	0.0928	0.1328
Guanyl-nucleotide exchange factor activity	GO:0005085	48	2	4.30E−02	0.0662	−0.6026
DNA repair	GO:0006281	256	29	4.63E−02	0.05	0.2181
Transmembrane transport	GO:0055085	235	25	4.69E−02	0.0869	0.4296
Exocytosis	GO:0006887	47	2	4.77E−02	0.6251	0.0608
Nuclear transport	GO:0051169	181	16	4.85E−02	0.1614	0.2033
Cellular bud	GO:0005933	241	26	4.92E−02	0.047	0.1037

a PCC, Pearson’s correlation coefficient following expression throughout the cell cycle (24).

### The Saccharomyces cerevisiae genomic positioning relationships are conserved across widely divergent fungal species.

**FIG 2:**
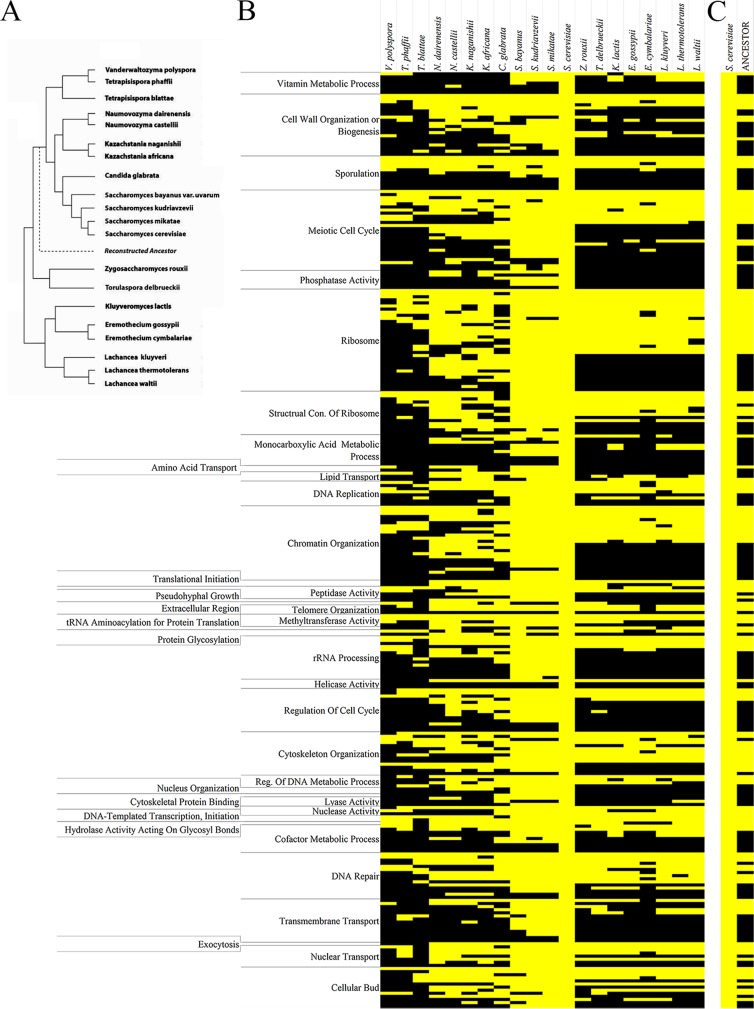
The functionally clustered genes are a combination of conserved, ancestral pairings and newly evolved species-specific pairings. The functionally clustered gene pairings from Saccharomyces cerevisiae were analyzed for conservation of the pairings that are seen in S. cerevisiae. The relationship of the species analyzed in this study is shown in panel A, and the levels of conservation are shown in divergent fungal lineages (B) and the last common ancestor before the whole-genome duplication event (C). The heat map depicts the conservation with either yellow (conservation of the pairing) or black (no conservation of the pairing).

In order to understand the evolutionary significance of these functional clustering relationships identified, we characterized the extent of conservation maintained across widely divergent fungal lineages. Starting with S. cerevisiae as a reference point, conservation of the same functional clusters was determined for every gene classification that demonstrated a significant (*P* < 0.05), nonrandom genomic distribution (Fig. 2). Our analysis focused on the conservation of the exact pairing relationship that exists in S. cerevisiae within each organism. Our analysis first began with the closely related yeasts S. mikatae, S. kudriavzevii, and S. bayanus, where we observed extensive conservation of the S. cerevisiae genomic arrangement in all three species. As we expanded our analysis to species with a greater evolutionary distance from S. cerevisiae, the yeasts *Candida glabrata*, Kazachstania africana, Kazachstania naganishii, Naumovozyma castelli, and Naumovozyma dairenensis, there continued to be large numbers of conserved clusters—although the levels are significantly lower than the more closely related yeast species (Fig. 2).

This analysis was expanded to the much more distantly related yeast species, including Tetrapisispora blattae, Tetrapisispora phaffii, Vanderwaltozyma polyspora, Zygosaccharomyces rouxii, Torulaspora delbrueckii, Kluyveromyces lactis, Eremothecium gossypii, Eremothecium cymbalariae, Lachancea kluyveri, Lachancea thermotolerans, and Lachancea waltii. Although there continued to be a drop-off in terms of the absolute levels of conservation of functional clusters as we analyzed species with greater and greater evolutionarily distances, we continued to observe extensive conservation of the groupings in all of the species analyzed in this study (Fig. 2B). When comparing the clustering conservation between S. cerevisiae and the last common ancestor before the yeast whole-genome duplication, it is clear that there is wide variation in the formation of the clustering relationships that are seen—some represent ancestral relationships and genome arrangements, and others are much newer in age (Fig. 2C).

There is also wide variation between the conservation within specific gene family classifications as well as across gene families. Some of the greatest levels of conservation are seen in the ribosome, rRNA processing, chromatin organization, meiotic cell cycle, and DNA repair gene families; however, in each family there are pairings that are not conserved beyond S. cerevisiae. Additionally, the statistical significance of a gene family’s genomic distribution was a poor predictor of conservation, as the three families with the most significant *P* values, the vitamin metabolic process (*P* = 9.061 × 10^−13^), cell wall organization or biogenesis (8.349 × 10^−10^), and sporulation (5.092 × 10^−07^) families, did not exhibit the greatest levels of conservation. In fact, the conservation of vitamin metabolic process gene pairings was among the weakest among the gene families.

## DISCUSSION

### The clustering of functionally related genes coordinates transcription within gene families.

The role of spatial positioning transcriptional regulation has been recognized in many contexts and species. In Drosophila melanogaster, the relocalization of genes from euchromatin to heterochromatic regions typically results in the silencing of the corresponding gene by “position effect variegation,” which is dependent on chromatin modification (25). The insertion of genes near the telomeres results in silencing called the “telomere proximal effect” (TPE). The TPE was characterized in S. cerevisiae and has been found in more complex eukaryotic organisms, including mice and human cells (26–28).

Likewise, promoters have been observed exerting transcriptional regulatory effects over long genomic distances in eukaryotes, which is conserved in species ranging from yeasts to humans (29). In human cells, the normal transcriptional activation of a single gene resulted in an increase of transcription throughout a chromosomal neighborhood, while in S. cerevisiae, long-distance transcriptional activation has been well characterized and is dependent on the Mediator complex (30–32). Our present work builds upon these observations, extends our understanding of adjacent-gene coregulation, and provides evidence that myriad gene families are organized into nonrandom clusters and that this distribution results in tighter transcriptional regulation.

This genomic arrangement was first observed in the RP and RRB families, but it was also observed in five other functionally related gene families. These five previously identified gene families had not been characterized transcriptionally, and four families exhibited a tighter transcriptional control for the clustered genes than the individually located members of each group. The sole outliers were the HSP genes, which may be due to mutually exclusive expression within the clusters under the conditions that we chose to analyze. This has been seen before as a mechanism regulating serine-responsive regulatory elements, where transcription of *SER3* is regulated by the adjacent transcript *SRG1* (33, 34). Thus, the functional clustering that we have seen may also facilitate the mutually exclusive expression of gene pairs in specific contexts.

Another related phenomenon was seen when studying the effects that the insertion of a reporter gene at various sites throughout the genome can have in S. cerevisiae. It was found the reporter frequently can disrupt the transcription of neighboring genes, which has been termed the “neighboring-gene effect”: this has been predicted to have resulted in the misannotation of 7 to 15% of gene functions in the yeast mutation libraries (35). Our work differs from this previous study in that we are characterizing the behavior of genes within their endogenous context under their own promoter. Rather than rely on reporters, our work further elucidates our understanding of the interplay occurring within specific genomic contexts—and is parsimonious with these previous studies.

### The transcriptional coordination of functionally clustered gene pairings is correlated with conservation of pairing.

This phenomenon was not an S. cerevisiae-specific phenomenon—our analysis provides support that transcriptional coordination via spatial positioning is conserved in S. paradoxus, S. mikatae, and S. kudriavzevii. Overall, the majority of the families of functionally related genes had a tighter transcriptional coregulation of the clustered pairings compared to the singleton members in each set, with the exception being the HSP and DDR genes seen in S. kudriavzevii. However, the individual responses of each family to specific stressors indicate that this is not an absolute; there is an increase in transcription seen in the clusters for specific stressors. It is interesting to speculate that one of the driving forces that could result in the formation of the clusters within a gene family could be driven, in part, by the transcriptional response-specific conditions—such as the ability of the nitrogen metabolism genes to respond to nitrogen depletion—and that certain functional groupings need tighter coregulation in order to properly manage cellular energy expenditures (e.g., the RPs).

One model is that the genomic rearrangements resulting in functional clusters are relatively passive in nature. One could envision that the clusters result from chance rearrangements whereby two functionally related genes end up directly adjacent to one another in a more permissive chromosomal region. Over time, the regulatory elements may evolve to exert their influence over the gene pairs leading to adjacent-gene coregulation. *cis* regulatory elements have been shown to exert their influence at significant genomic distances, and there is wide variability in the permissive nature of different genomic regions (29, 30). Such a model could offer an explanation as to why functional clusters can be observed in so many diverse gene families—each of which has its own specific *cis* regulatory sequences and *trans* factors.

Such a model could also explain the fact that although the exact functional clusters are not conserved throughout all fungi, there are similar absolute levels of clustering of RP and RRB genes that are seen in Candida albicans and Schizosaccharomyces pombe (17). Likewise, a similar, nonrandom genomic distribution of both the RP and RRB genes exists in many higher eukaryotic species, suggesting this process may be a fundamental mechanism that facilitates efficient transcriptional regulation of functionally related genes (20). This model could lend insight into why there have been domains of coexpressed genes seen in many species in addition to S. cerevisiae, including Caenorhabditis elegans, Drosophila melanogaster, Arabidopsis thaliana, and Danio rerio (21, 36–39). This process could arise stochastically, although once it occurs, it is possible there is selection on the clustered grouping.

### A significant fraction of functionally related gene families are found in clusters and conserved in diverse fungal lineages.

In light of the apparent significance that the nonrandom genomic distribution of functionally related genes plays in the coordination of transcription within the few regulons characterized, we sought a systematic way to identify the prevalence of this occurrence. The use of GO Slim categorizations represented an easily identifiable series of groupings transferrable to many species. The 140 categorizations represented a wide swath of genes that function in myriad cellular and molecular processes. It was surprising that 27% of these categorizations exhibited a nonrandom genomic distribution as functionally related gene clusters. Due to the many different processes in which these genes function, we assessed it was more prudent to characterize the transcriptional regulation throughout the cell cycle—as some of these gene families would not necessarily yield a robust transcriptional response during a stress response. In all cases, every ontological classification exhibited a positive transcriptional response, albeit some were much stronger than others. Many of these (approximately 74%) exhibited a higher PCC when found in functional clusters than when found in isolation as singletons. While this is surprising (e.g., not all helicases would be under the same transcriptional regulation), it is consistent with previous reports and will warrant future study to further dissect the mechanisms that underlie this regulation.

Our analysis focused exclusively on the conservation of the exact pairing relationships seen in S. cerevisiae only. The rationale for this was guided by a previous analysis that found the incidence of S. cerevisiae functional pairings with a new member of the same family was extremely low—almost negligible (18). The extent of conservation of the functional clusters in diverse fungi, combined with previous reports, suggests that adjacent-gene coregulation plays a greater role in transcriptional regulation in eukaryotes than previously appreciated (17, 20).

### Conclusion.

While it has been observed that neighboring genes influence the expression of each other within a finite window, it appears that many species of fungi may exploit this phenomena to help regulate functionally related gene sets via their spatial positioning throughout the genome. This genomic arrangement represents one level of transcriptional control that helps to maintain coordinated levels of gene expression. Thus, the phenomenon of adjacent-gene coregulation that occurs via functional clustering may be much more widespread than previously appreciated.

## MATERIALS AND METHODS

### Gene expression analysis of transcription profiles.

Microarray expression profiles were downloaded for the following species of yeast: S. cerevisiae, S. paradoxus, S. mikatae, and S. kudriavzevii (40). The specific data sets corresponded to five environmental and nutritional stressors: heat shock exposure at 37°C, oxidative stress to 0.3 mM H_2_O_2_, DNA damage response from exposure to 0.02% MMS, nitrogen starvation from omitting ammonium sulfate from the growth medium, and carbon source transition from 2% glucose to 3% glycerol (GEO accession no. GSE3406). The data analyzed for synchronized cycling cells followed a time course upon the release from α-factor synchronization (GEO accession no. GDS38) in Saccharomyces cerevisiae (24). We identified the genes for a given regulon in the data set and multiple replicates, and time points were averaged together in order to give a single time course for analysis for each condition and for each species.

### Calculating the average pairwise Pearson’s correlation coefficient from transcription profiles.

The transcriptional similarity between two genes was calculated as previously described (41). To calculate the Pearson’s correlation coefficient (PCC) between two genes, *X* and *Y*, across a series of *N* conditions:

$$S\left(X,Y\right)=\frac{1}{N}\sum_{i=1}^{N}\left(\frac{X_{i}-X_{\text{offset}}}{\phi_{X}}\right)\left(\frac{Y_{i}-Y_{\text{offset}}}{\phi_{Y}}\right)$$

where

$$\phi_{G}=\sqrt{\sum_{i=1}^{N}\frac{{\left(G_{i}-G_{\text{offset}}\right)}^{2}}{N}}$$

*G*_offset_ was set to the reference state in each data set. The PCC scores for the unpaired genes are calculated from the average of every possible pairing partner for every possible unpaired gene. *P* values were determined by bootstrapping with replacement by taking at least 10,000 random groupings of genes (the same size as the paired subset) and determining the average PCC score for that grouping. We decided to use this number empirically, as the frequency plots did not change significantly between 10,000 and 100,000 iterations for either the ribosomal protein genes (Fig. S2A) or the carbohydrate metabolism genes (Fig. S2B). This indicated that we had sampled all possible combinations in entirety. To ensure that there was no bias in the selections, the frequency of every particular gene was reported and plotted to ensure equal representation of every gene within each set (see Fig. S3 in the supplemental material).

10.1128/mSphere.00220-18.3 FIG S3  Frequency histograms for the bootstrapping analysis stabilize at 10,000 iterations. Shown is the transcriptional correlation for every possible functional clustering arrangement that could have arisen through the use of bootstrapping with replacement for both the RP regulon (A) and the CM gene families (B). The analysis was run 1,000, 10,000, and 100,000 times for each gene family, and our analysis was based on the stabilization of the histogram shape that was seen at 10,000 iterations. Download FIG S3, TIF file, 0.5 MB. Copyright © 2018 Eldabagh et al. 2018 Eldabagh et al. This content is distributed under the terms of the Creative Commons Attribution 4.0 International license.

### Determining the conservation of gene pairing relationships in divergent fungal lineages.

The functional clusters of adjacent-gene pairs identified in S. cerevisiae were compared with a divergent selection of related yeast species to characterize the extent of conservation of spatial positioning. The conservation of the clusters was determined by homology and synteny by using the Yeast Gene Order Browser focusing on conservation in Saccharomyces mikatae, Saccharomyces kudriavzevii, Saccharomyces bayanus, *Candida glabrata*, Kazachstania africana, Kazachstania naganishii, Naumovozyma castelli, Naumovozyma dairenensis, T. blattae, T. phaffii, V. polyspora, Z. rouxii, T. delbrueckii, K. lactis, E. gossypii, E. cymbalariae, L. kluyveri, L. thermotolerans, and L. waltii, as well as the reconstructed ancestor that existed prior to whole-genome duplication (42–44).

### Calculating the statistical significance of gene adjacency.

The statistical significance for the genomic distribution of a functionally related gene family was calculated by determining the binomial probability as previously described (17). The chance probability that there would be *j* adjacent genes within a regulon of size *M* genes is

$$1-\overset{j}{\underset{k=0}{\sum }}\left(\frac{M!}{k!\left(M-k\right)!}\right)\left(P^{k}{\left(1-P\right)}^{M-k}\right)$$

where

$$P=\left(\frac{M}{N}\right)\left(2-\frac{M}{N}\right)$$

*N* is the total number of genes present within S. cerevisiae (total number of genes after deduction of dubious open reading frames). The functional *P* values were then calculated in Mathematica.

### Data availability.

Microarray expression profiles are available from the Gene Expression Omnibus for the environmental and nutritional stressors (GEO accession no. GSE3406) and throughout the cell cycle (GEO accession no. GDS38). Software written by the authors and used to perform PCC analysis is freely distributed under a Gnu Public License and may be accessed at https://github.com/FoleyLab/Pearsons_mShere.

10.1128/mSphere.00220-18.4 FIG S4  The frequency of selection of every gene was reported for each family and plotted to ensure that there was no bias in the selections. To ensure that there was no bias in the selection of any particular gene in our algorithm, the frequency of selection for every gene was reported and plotted as a histogram. Download FIG S4, TIF file, 0.4 MB. Copyright © 2018 Eldabagh et al. 2018 Eldabagh et al. This content is distributed under the terms of the Creative Commons Attribution 4.0 International license.
