# Supplementary figures and images for: Systematic Identification, Characterization, and Conservation of Adjacent-Gene Coregulation in the Budding Yeast Saccharomyces cerevisiae
Source: mSphere. 2018 Jun 13;3(3):e00220-18. doi: 10.1128/mSphere.00220-18 (PMC6001612; doi:10.1128/mSphere.00220-18)

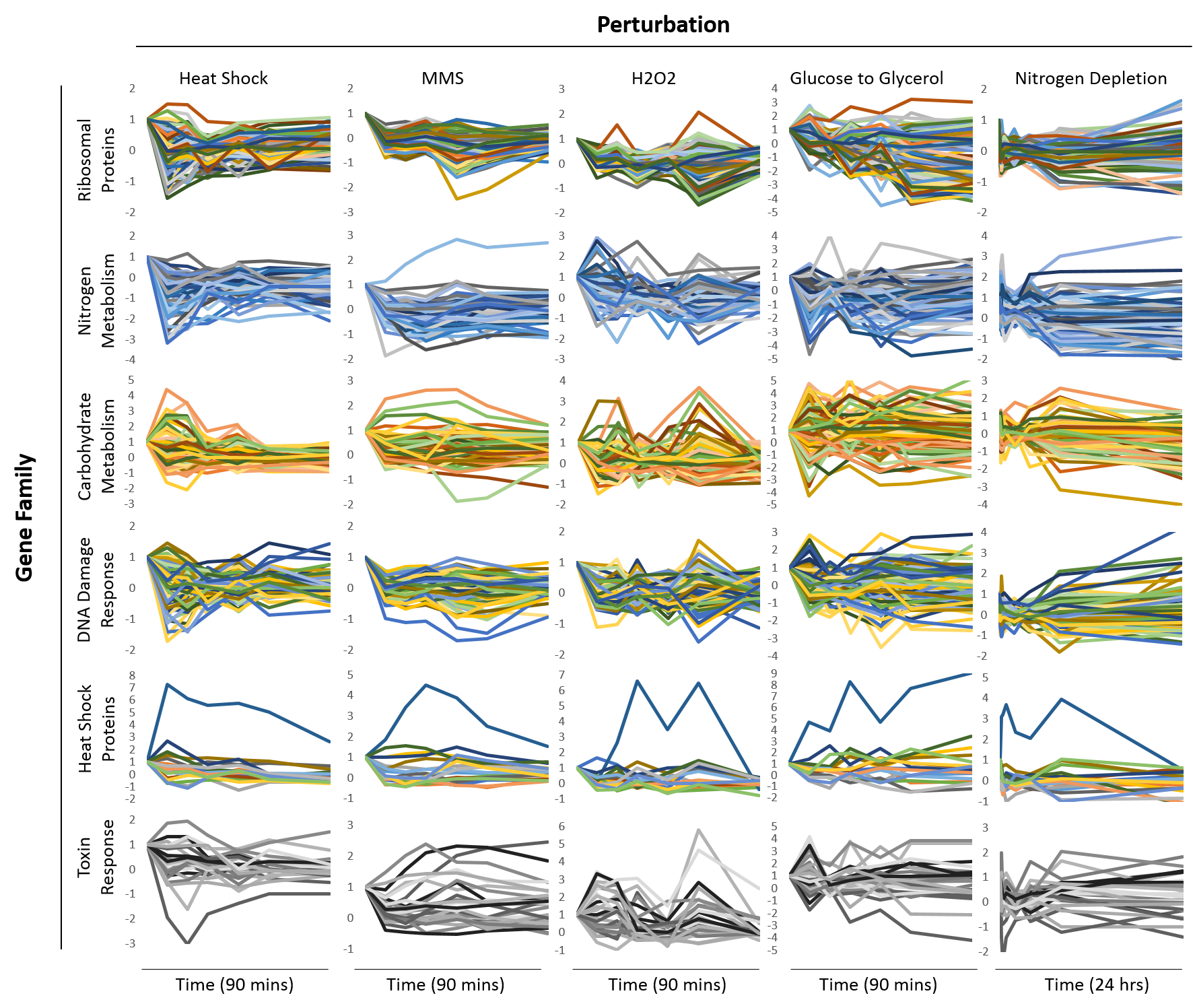

Supplement: FIG S1 [file sph003182565sf1.tif]

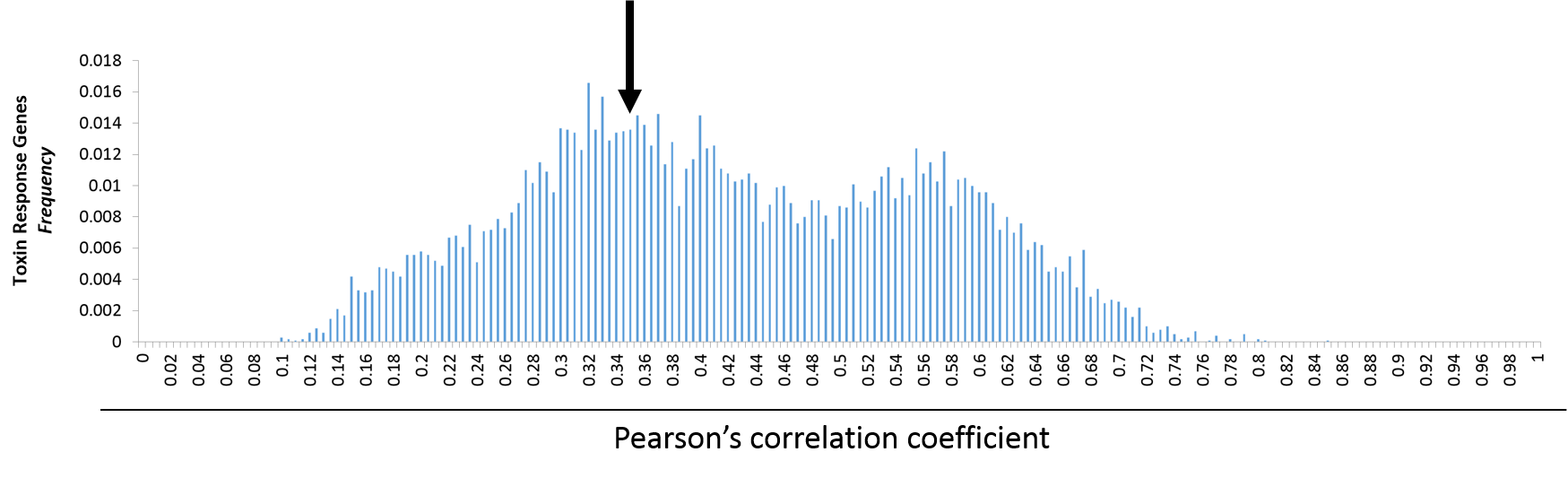

Supplement: FIG S2 [file sph003182565sf2.tif]

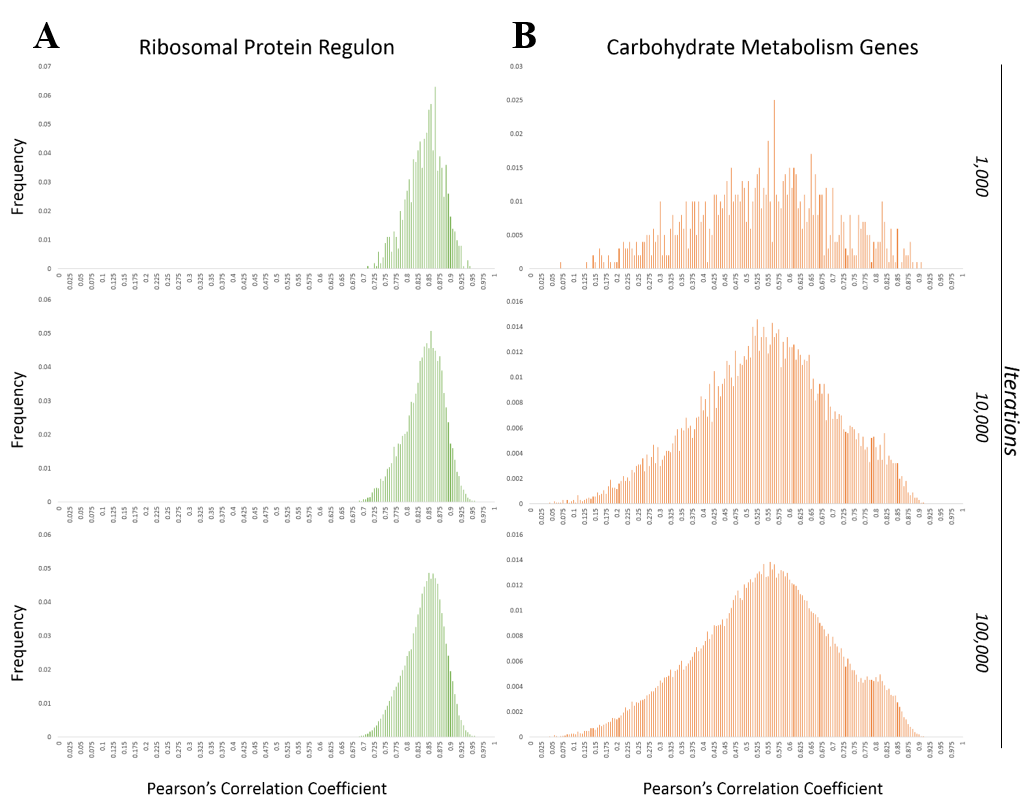

Supplement: FIG S3 [file sph003182565sf3.tif]

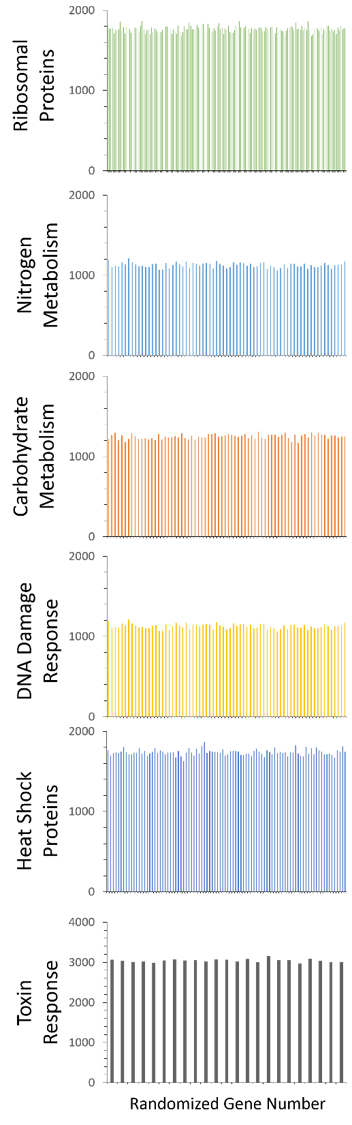

Supplement: FIG S4 [file sph003182565sf4.tif]
