# Supplementary material for: Systematic Identification, Characterization, and Conservation of Adjacent-Gene Coregulation in the Budding Yeast Saccharomyces cerevisiae
Source: mSphere. 2018 Jun 13;3(3):e00220-18. doi: 10.1128/mSphere.00220-18 (PMC6001612; doi:10.1128/mSphere.00220-18)
Supplement: TABLE S1 [file sph003182565st1.docx]

| Supplemental Table #1: Functionally Related Gene Families that Exhibit a Random Genomic Distribution in *S. cerevisiae* (p>0.05) | | | | |
| --- | --- | --- | --- | --- |
| **Description** | **G. O. number** | **Gene set size** | **# adjacent genes** | **P-value** |
| Protein Transporter Activity | GO:0008565 | 51 | 2 | 0.0579 |
| Nucleus | GO:0005634 | 2032 | 1194 | 0.0602 |
| Cellular Response to DNA Damage Stimulus | GO:0006974 | 307 | 39 | 0.0613 |
| Transferase Activity | GO:0016740 | 790 | 216 | 0.0618 |
| mRNA Processing | GO:0006397 | 163 | 13 | 0.0631 |
| Protein Maturation | GO:0051604 | 52 | 2 | 0.0635 |
| ATPase Activity | GO:0016887 | 265 | 30 | 0.0636 |
| Plasma Membrane | GO:0005886 | 396 | 61 | 0.0691 |
| Nucleolus | GO:0005730 | 252 | 27 | 0.0767 |
| Response to Starvation | GO:0042594 | 56 | 2 | 0.0898 |
| Cytokinesis | GO:0000910 | 84 | 4 | 0.0902 |
| Mitochondrion Organization | GO:0007005 | 266 | 29 | 0.0994 |
| Mitotic Cell Cycle | GO:0000278 | 319 | 40 | 0.1065 |
| Signaling | GO:0023052 | 241 | 24 | 0.1117 |
| Chromatin Binding | GO:0003682 | 111 | 6 | 0.1221 |
| Isomerase Activity | GO:0016853 | 60 | 2 | 0.1222 |
| Mitochondrial Envelope | GO:0005740 | 355 | 48 | 0.1246 |
| Protein Binding, Bridging | GO:0030674 | 62 | 2 | 0.1407 |
| Chromosome Segregation | GO:0007059 | 203 | 17 | 0.1475 |
| GTPase Activity | GO:0003924 | 63 | 2 | 0.1505 |
| Response to Osmotic Stress | GO:0006970 | 92 | 4 | 0.1574 |
| Ion Transport | GO:0006811 | 263 | 27 | 0.1599 |
| Carbohydrate Metabolic Process | GO:0005975 | 198 | 16 | 0.1632 |
| Cellular Amino Acid Metabolic Process | GO:0006520 | 200 | 16 | 0.1833 |
| Oxidoreductase Activity | GO:0016491 | 307 | 35 | 0.2035 |
| Site of Polarized Growth | GO:0030427 | 262 | 26 | 0.2045 |
| transcription from RNA polymerase I promoter | GO:0006360 | 68 | 2 | 0.2052 |
| regulation of organelle organization | GO:0033043 | 283 | 30 | 0.2064 |
| Organelle Fusion | GO:0048284 | 121 | 6 | 0.2228 |
| Organelle Fission | GO:0048285 | 304 | 34 | 0.2229 |
| Unfolded Protein Binding | GO:0051082 | 70 | 2 | 0.2294 |
| Nucleobase-Containing Small Molecule Metabolic Process | GO:0055086 | 198 | 15 | 0.2379 |
| response to heat | GO:0009408 | 73 | 2 | 0.2681 |
| RNA Modification | GO:0009451 | 102 | 4 | 0.2731 |
| Enzyme Regulator Activity | GO:0030234 | 234 | 20 | 0.2808 |
| NucleotidyltransferaseActivity | GO:0016779 | 78 | 2 | 0.3374 |
| Peptidyl-Amino Acid Modification | GO:0018193 | 151 | 8 | 0.3561 |
| RNA Catabolic Process | GO:0006401 | 132 | 6 | 0.3687 |
| DNA Binding | GO:0003677 | 396 | 53 | 0.3729 |
| Proteolysis Involved in Cellular Protein Catabolic Process | GO:0051603 | 216 | 16 | 0.3874 |
| Regulation of Protein Modification Process | GO:0031399 | 111 | 4 | 0.3996 |
| Regulation of Transport | GO:0051049 | 85 | 2 | 0.4410 |
| Hydrolase Activity | GO:0016787 | 830 | 218 | 0.4621 |
| Enzyme Binding | GO:0019899 | 87 | 2 | 0.4711 |
| Protein Folding | GO:0006457 | 93 | 2 | 0.5603 |
| Golgi apparatus | GO:0005794 | 199 | 12 | 0.5649 |
| Cytoskeleton | GO:0005856 | 200 | 12 | 0.5795 |
| Transcription Factor Activity, Protein Binding | GO:0000988 | 124 | 4 | 0.5921 |
| Nucleic Acid Binding Transcription Factor Activity | GO:0001701 | 167 | 8 | 0.5922 |
| DNA recombination | GO:0006310 | 185 | 10 | 0.5962 |
| Ligase Activity | GO:0016874 | 98 | 2 | 0.6312 |
| Cellular Ion Homeostasis | GO:0006873 | 128 | 4 | 0.6478 |
| Ribosomal Large Subunit Biogenesis | GO:0042273 | 104 | 2 | 0.7092 |
| Response to Oxidative Stress | GO:0006979 | 105 | 2 | 0.7213 |
| tRNA Processing | GO:0008033 | 107 | 2 | 0.7446 |
| Membrane Fusion | GO:0061025 | 109 | 2 | 0.7667 |
| RNA splicing | GO:0008380 | 138 | 4 | 0.7702 |
| Cytoplasmic Vesicle | GO:0031410 | 237 | 15 | 0.7744 |
| Histone Modification | GO:0016570 | 114 | 2 | 0.8167 |
| RNA Binding | GO:0003723 | 384 | 42 | 0.8179 |
| Vesicle Organization | GO:0016050 | 168 | 6 | 0.8384 |
| Cell Cortex | GO:0005938 | 147 | 4 | 0.8547 |
| Nucleobase-Containing Compound Transport | GO:0015931 | 151 | 4 | 0.8842 |
| Kinase Activity | GO:0016301 | 542 | 84 | 0.8861 |
| Protein Targeting | GO:0006605 | 306 | 24 | 0.8900 |
| Conjugation | GO:0000746 | 124 | 2 | 0.8936 |
| Membrane | GO:0016020 | 1629 | 749 | 0.9077 |
| Ribosomal Small Subunit Biogenesis | GO:0042274 | 130 | 2 | 0.9262 |
| Vacuole | GO:0005773 | 470 | 60 | 0.9320 |
| Lipid Metabolic Process | GO:0006629 | 297 | 20 | 0.9601 |
| Mitochondrion | GO:0005739 | 1129 | 362 | 0.9643 |
| mRNA Binding | GO:0003729 | 172 | 4 | 0.9721 |
| Golgi vesicle transport | GO:0048193 | 191 | 2 | 0.9997 |
| Cytoplasm | GO:0005737 | 3990 | 3496 | 1.0000 |
| Cell Morphogenesis | GO:0000902 | 29 | 0 | n.s. |
| Cellular Respiration | GO:0045333 | 83 | 0 | n.s. |
| DNA-templated transcription, elongation | GO:0006354 | 96 | 0 | n.s. |
| DNA-templated transcription, termination | GO:0006353 | 43 | 0 | n.s. |
| Endomembrane System | GO:0012505 | 22 | 0 | n.s. |
| Endosomal Transport | GO:0016197 | 102 | 0 | n.s. |
| Generation of Precursor Metabolites and Energy | GO:0006091 | 154 | 0 | n.s. |
| Histone Binding | GO:0042393 | 29 | 0 | n.s. |
| Invasive Growth in Response to Glucose Limitation | GO:0001403 | 59 | 0 | n.s. |
| Membrane Invagination | GO:0010324 | 74 | 0 | n.s. |
| Microtubule Organizing Center | GO:0005815 | 73 | 0 | n.s. |
| Oligosaccharide Metabolic Process | GO:0009311 | 2 | 0 | n.s. |
| Organelle Inheritance | GO:0048308 | 60 | 0 | n.s. |
| Peroxisome | GO:0005777 | 76 | 0 | n.s. |
| Peroxisome Organization | GO:0007031 | 26 | 0 | n.s. |
| Protein Alkylation | GO:0008213 | 50 | 0 | n.s. |
| Protein Dephosphorylation | GO:0006470 | 49 | 0 | n.s. |
| Protein Lipidation | GO:0006497 | 45 | 0 | n.s. |
| Regulation of Translation | GO:0006417 | 103 | 0 | n.s. |
| Ribosomal Subunit Export from Nucleus | GO:0000054 | 46 | 0 | n.s. |
| Ribosome Assembly | GO:0042255 | 58 | 0 | n.s. |
| rRNA Binding | GO:0019843 | 31 | 0 | n.s. |
| Signal Transducer Activity | GO:0004871 | 38 | 0 | n.s. |
| snoRNA Processing | GO:0043144 | 44 | 0 | n.s. |
| Transcription Factor Binding | GO:0008134 | 70 | 0 | n.s. |
| Translation Factor Activity, RNA binding | GO:0008135 | 1 | 0 | n.s. |
| Ubiquitin-like Protein Binding | GO:0032182 | 44 | 0 | n.s. |
| Vacuole Organization | GO:0007033 | 88 | 0 | n.s. |
